# Successful Treatment of a Rapidly Enlarging Mandibular Aneurysmal Bone Cyst With Sclerotherapy and Intralesional Curettage

**DOI:** 10.7759/cureus.78256

**Published:** 2025-01-30

**Authors:** Katsuhiko Sakai, Masaki Minabe, Kasumi Hata, Koki Kamemoto, Koichi Masuda, Kazuhiko Hashimoto, Takeshi Nomura, Nobuyuki Matsuura

**Affiliations:** 1 Oral Medicine and Hospital Dentistry, Tokyo Dental College, Ichikawa, JPN; 2 Oral Medicine, Oral and Maxillofacial Surgery, Ichikawa General Hospital, Tokyo Dental College, Ichikawa, JPN; 3 Oral Oncology, Oral Maxillofacial Surgery, Tokyo Dental College, Ichikawa, JPN; 4 Radiology, Ushiku Aiwa General Hospital, Ushiku, JPN; 5 Pathology and Laboratory Medicine, Ichikawa General Hospital, Tokyo Dental College, Ichikawa, JPN

**Keywords:** aneurysmal bone cyst, intralesional curettage, mandible tumor, polidocanol, sclerotherapy

## Abstract

An aneurysmal bone cyst (ABC) is a benign bone lesion primarily found in the metaphysis of long bones or spine, with rare occurrences in the head and neck region. We report the case of a 17-year-old female patient with a rapidly enlarging mandibular ABC. Initial imaging and biopsy confirmed the diagnosis of ABC, revealing a blood-filled lesion with multinucleated giant cells and no solid components. Owing to the lesion’s size and rapid growth, hemimandibulectomy was initially considered but postponed due to concerns about aesthetic and functional outcomes. The patient underwent percutaneous sclerotherapy with polidocanol, followed by intralesional curettage. Post-treatment evaluations indicated complete lesion resolution and improved facial symmetry with no recurrence at 36 months. This case highlights the potential benefits of combining polidocanol sclerotherapy and intralesional curettage for the treatment of jaw ABCs. Further research is needed to determine the optimal application and follow-up of this approach.

## Introduction

An aneurysmal bone cyst (ABC) is a benign, blood-filled bone lesion that accounts for 1% of all bone tumors and most frequently occurs in the second decade of life [1-3]. It is typically found in the metaphysis of the long bones or spine; meanwhile, only 2-6% of ABCs occur in the head and neck region, most frequently in the mandible [4,5]. ABCs are often associated with bone destruction and can grow rapidly [6]. The primary treatment for ABCs in the head and neck is surgical excision [4,5]. For large mandibular lesions, complete resection through mandibulectomy is performed, though concerns about aesthetic and functional complications persist [4,7-9]. Recently, the use of minimally invasive non-surgical treatments have been reported, with percutaneous sclerotherapy using polidocanol showing promising results [10-13]. However, only a few studies have explored non-surgical therapies for head and neck lesions. We encountered a rapidly enlarging ABC in the mandible of a teenage girl. Treatment with polidocanol-based sclerotherapy and intralesional curettage facilitated the preservation of the jawbone.

## Case presentation

**Figure 1:**
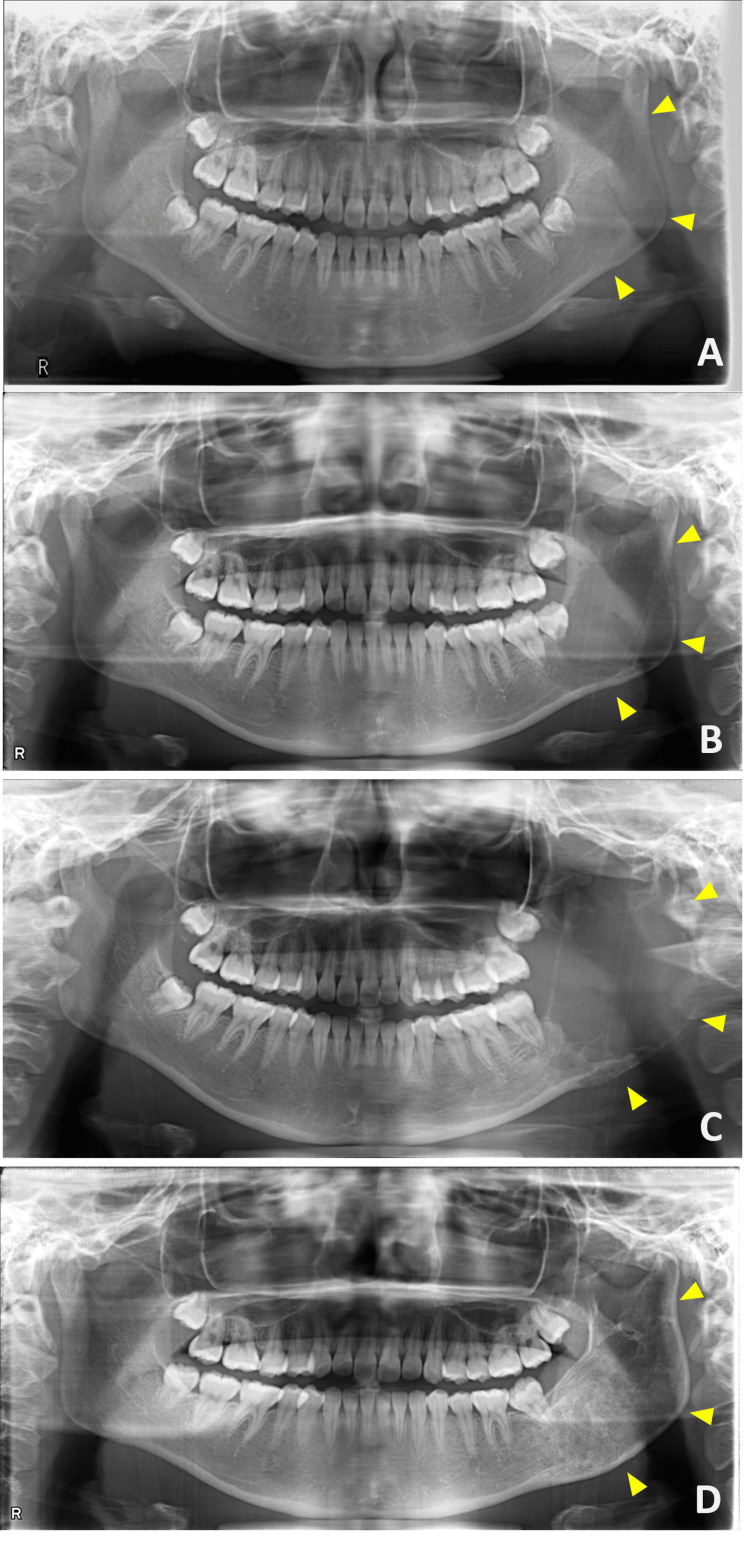
Orthopantomogram X-ray (A) Eight months prior to the initial visit, no abnormalities were observed. (B) At the initial visit, a radiolucent area was observed in the left mandibular ramus. (C) Before sclerotherapy, the radiolucent area had increased in size. (D) At 24 months post operation, the lesion had resolved, with evidence of bone regeneration. The arrows indicate the mandibular ramus before and after the onset of the lesion, as well as after healing.

A 17-year-old female patient was referred to the department of Oral Medicine, Oral and Maxillofacial Surgery, Ichikawa General Hospital, Tokyo Dental College by a general practitioner on December 2, 2020 with a chief complaint of swelling in the left cheek area. Eight months prior to her initial visit, she experienced discomfort in the left mandibular gingiva; her general practitioner performed an orthopantomogram, which showed no abnormalities (Figure 1A). One month prior to her initial visit, she noticed a gradually progressing swelling in the left cheek. She had no relevant medical history, family history, allergies, or a history of head or neck trauma or surgeries.

**Figure 2:**
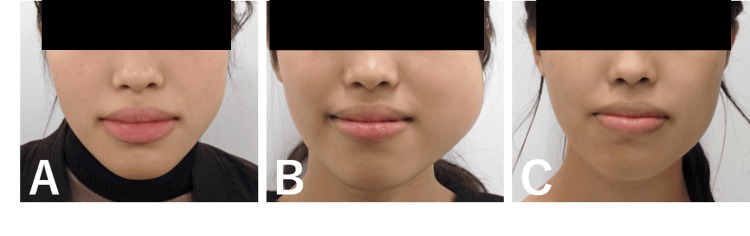
Photographs of the patient’s face (A) Initial presentation showing mild swelling in the left cheek area. (B) Increased swelling in the left cheek area compared with the initial presentation before sclerotherapy. (C) At 24 months post operation, the swelling in the left cheek area had subsided, and facial symmetry had improved.

**Figure 3:**
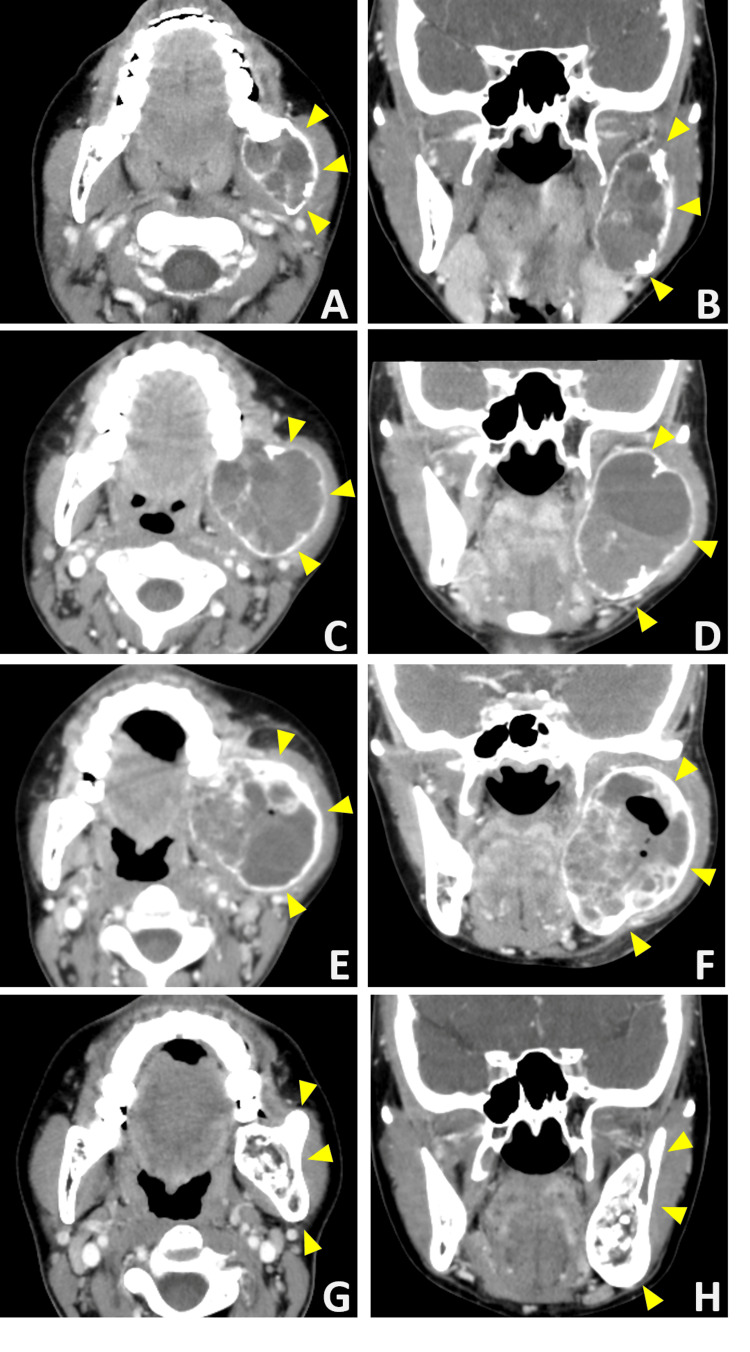
Contrast-enhanced CT images (A,B) At the initial visit, a radiolucent area measuring up to 50×30 mm was observed in the left mandibular ramus. (C,D) The lesion size had increased to a maximum of 65×40 mm before sclerotherapy. (E,F) After sclerotherapy, the lesion showed mild reduction with marginal calcification. (G,H) At 24 months post operation, the lesion had resolved, with evidence of bone regeneration. The arrows indicate the lesion and the healed mandibular ramus.

**Figure 4:**
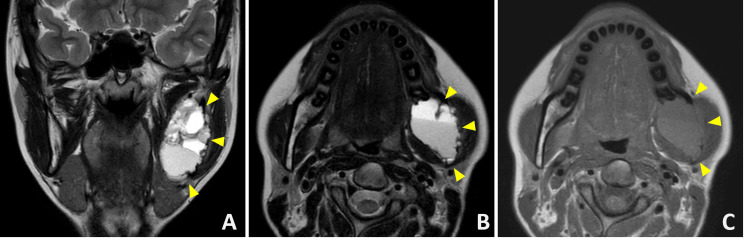
Preoperative contrast-enhanced MRIs (A,B) T1-weighted images showing a multilocular lesion with high signal intensity. (C) T2-weighted image indicating fluid retention within the lesion. The arrows indicate the lesion.

Upon initial examination, mild swelling was observed in the left cheek (Figure 2A). Intraoral examination revealed redness and swelling around the left mandibular second molar gingiva. Another orthopantomogram revealed a radiolucent area in the left mandibular ramus (Figure 1B). Meanwhile, contrast-enhanced CT showed a bone-lytic lesion measuring 50×30 mm in diameter in the left mandibular ramus (Figures 3A, 3B). Contrast-enhanced MRI revealed a multilocular lesion without significant solid components. The T2-weighted images indicated fluid retention, while the T1-weighted images suggested the presence of hemorrhage with high signal intensity (Figure 4).

Based on the imaging findings, an ABC was highly suspected, with differential diagnoses including osteosarcoma, ameloblastoma, and odontogenic keratocyst. Under local anesthesia using 3.6 mL of 2% lidocaine with 1:80,000 epinephrine, the left mandibular wisdom tooth was extracted, and a biopsy was performed. The lesion was filled with blood, with no evident solid components. Hence, the cyst wall was sampled. During the procedure, venous bleeding was noted and controlled using an absorbable hemostat (oxidized regenerated cellulose).

**Figure 5:**
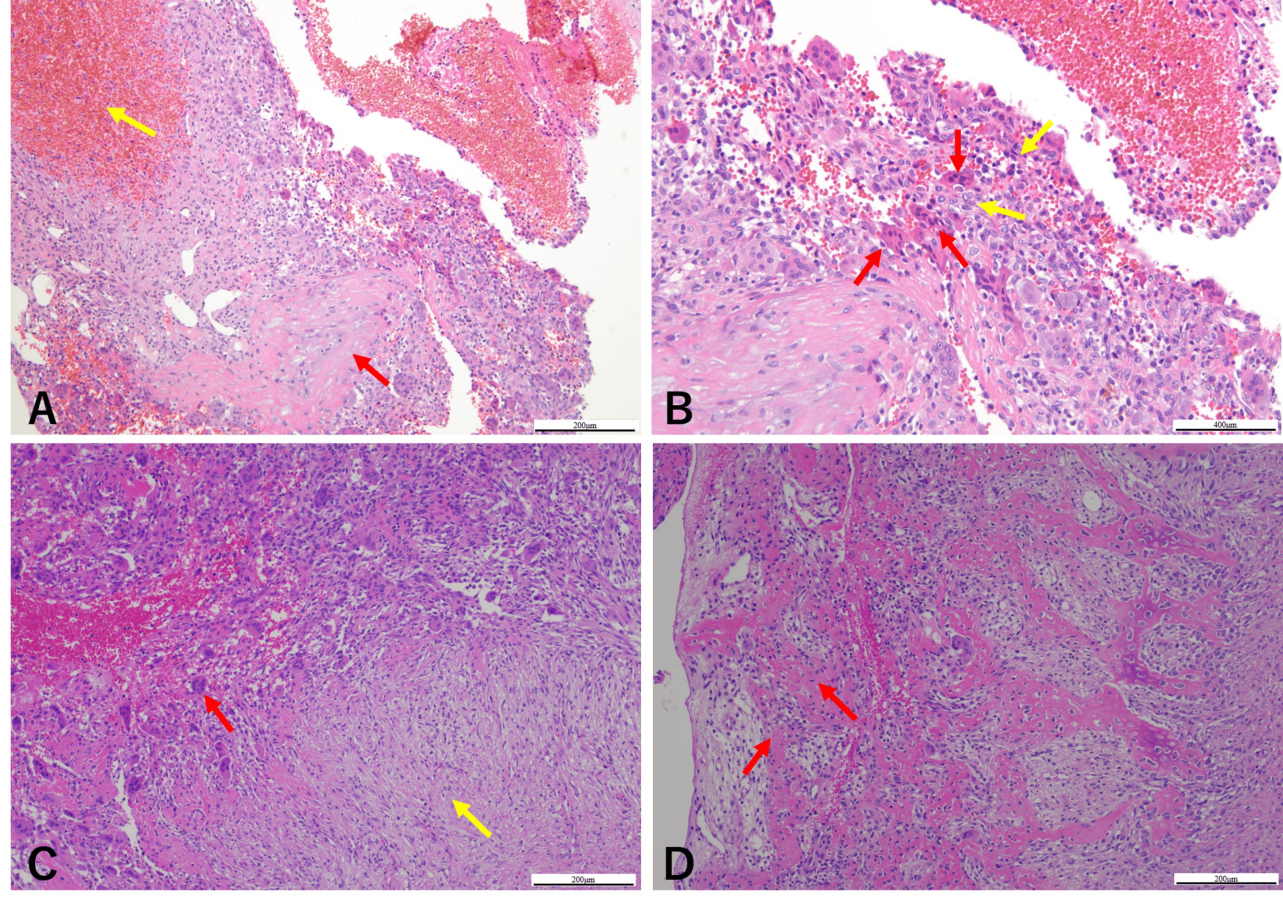
Histopathological findings (HE-stained tissue) (A)The biopsy tissue showed cyst-like connective tissue with abundant cellular components (red arrow) and hemorrhagic areas (yellow arrow) (×100). (B) The lumen contained numerous multinucleated giant cells (red arrows) and proliferating mononuclear cells (yellow arrows) (×200). (C) In the excised tissue after sclerotherapy, a few giant cell lesions similar to those observed in the biopsy were observed (red arrow), but the majority consisted of proliferating fibrous connective tissues (yellow arrow) (×100). (D) Active bone formation was observed in the area adjacent to the mandible (red arrows) (×100). HE: Hematoxylin and eosin

Histopathological examination using hematoxylin and eosin (HE) staining revealed a cyst wall-like connective tissue with hemorrhagic areas. The lumen contained numerous multinucleated giant cells and proliferating mononuclear cells (Figures 5A, 5B). These findings were consistent with the features of a giant cell lesion and supported the diagnosis of ABC, considering the clinical presentation. Fluorescence in situ hybridization (FISH) analysis showed no ubiquitin-specific protease 6 (USP6) rearrangement.

The cortical bone was thinned, and the lesion had become fragile, making intralesional curettage appear challenging. Given the size of the lesion, a hemimandibulectomy was considered necessary. However, concerns regarding the impact on both aesthetics and function were raised. Therefore, a provisional observation period was established, during which a drain was inserted. Five months after the initial consultation, the lesion had enlarged to 65×40 mm, becoming more exposed in the oral cavity and causing pain (Figures 1C, 2B, 3C, 3D). To avoid hemimandibulectomy, sclerotherapy with polidocanol was performed. The decision was made after consultations with the divisions of Oral Medicine, Oral Maxillofacial Surgery, and Radiology. Since sclerotherapy with polidocanol is considered off-label use in Japan, both the patient and their guardian were thoroughly informed about the procedure, including its potential benefits, risks, and off-label status. Written informed consent was obtained from both the patient and the guardian. A foam sclerosant composed of 1% polidocanol and air was injected into the lesion. The initial injection was administered using an intraoral approach, followed by an extraoral approach two months later. Post-treatment contrast-enhanced CT showed a mild reduction in lesion size with marginal calcification (Figures 3E, 3F). However, an infection subsequently developed, leading to pus discharge from the oral cavity.

**Figure 6:**
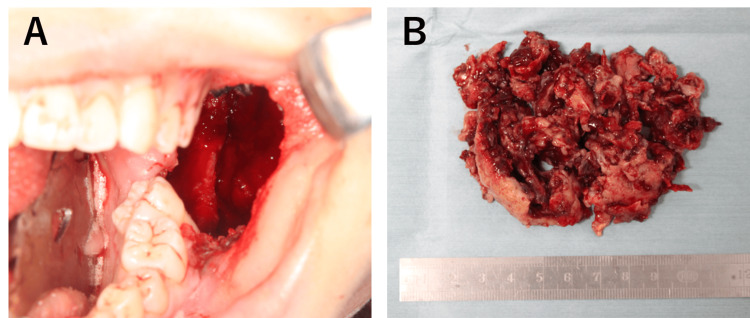
Clinical photographs (A) Intraoral view during surgery. (B) The resected lesion.

While restarting sclerotherapy after inflammation subsided was considered, the patient expressed concerns about prolonged treatment due to her academic commitments. Given the enhanced calcification surrounding the lesion, we determined that intralesional curettage was a viable option, which was performed under general anesthesia. An intralesional curettage of the lesion was performed under general anesthesia. The lesion was excised from the surrounding bone via an intraoral approach (Figure 6). Although the lesion was highly hemorrhagic, local hemostasis was achieved with an absorbable hemostat (oxidized regenerated cellulose), similar to the biopsy procedure. The estimated blood loss was approximately 800 mL. The wound was left open and packed with gauze, which was changed regularly. Complete wound closure was confirmed after two months. Histopathological examination of the HE-stained excised specimen showed a few giant cell lesions similar to those observed in the biopsy. However, the majority comprised proliferating fibrous connective tissues. Notably, active bone formation was observed in the area adjacent to the mandible (Figures 5C, 5D). Postoperatively, the patient underwent regular follow-ups and X-ray examinations. An orthopantomogram and CT scan conducted 24 months post-surgery confirmed the complete resolution of the lesion (Figures 1D, 3G, 3H). Additionally, facial symmetry was improved (Figure 2C). At 36 months post operation, there has been no recurrence.

## Discussion

The pathophysiology of ABC has been the subject of various discussions. Previous studies proposed that ABC may represent a reactive lesion resulting from localized vascular disturbances or abnormal reparative processes following trauma [2,3]. In Head and Neck Tumours: WHO Classification of Tumours, 5th Edition, Volume 9, WHO defines ABC as a cystic or multicystic expansile osteolytic neoplasm comprising spaces filled with blood, separated by fibrous septa, and containing osteoclast-type giant cells [14]. FISH has shown that 69% of patients with ABC exhibit USP6 rearrangement, supporting the neoplastic nature of ABC [15]. However, in patients with ABC in the jawbone, only 40% exhibit USP6 rearrangement [16]. This disparity suggests potential differences in the pathophysiology of ABC in the jawbone compared with other anatomical locations. In our case, although the lesion was pathologically and morphologically consistent with an ABC, USP6 rearrangement was not observed.

Moreover, despite being classified as a benign tumor, ABC is known for its aggressive growth tendency, which frequently causes pathological fractures [6,17]. The primary symptom is the development of a mass, which may or may not be accompanied by tenderness [4]. In the head and neck region, the average size of an ABC at the time of detection is reported to be 41.4 mm [5]. In our case, the patient presented with a non-tender mass, which measured 50 mm at the time of discovery. Notably, an orthopantomogram taken eight months prior to the initial visit did not show any mass, indicating a rapid growth tendency. The mass continued to grow even after the biopsy, reaching a maximum size of 65 mm. These observations suggest that due to the rapid growth tendency of ABC, it is crucial to diagnose it accurately and intervene at an early stage.

The most common treatment option for ABC is intralesional curettage, which can be performed with or without bone grafting. Adjuvant therapies, including phenol, cryotherapy, and argon beam coagulation, are utilized to reduce the risk of recurrence [3,12]. A review by Bavan et al. reported a failure or recurrence rate of 14.4% for intralesional curettage, with rates varying from 0% to 40% across different studies [12]. Although intralesional curettage is relatively less invasive, treatment failure and recurrence remain significant issues. A more aggressive surgical option is en bloc resection. While en bloc resection showed successful outcomes with the lowest recurrence rates, it is more invasive with higher morbidity and adverse effects associated with the procedure (bleeding, growth disturbances, infection risk) [1,2].

Surgical treatment is generally the first-line treatment for ABC occurring in the head and neck, with most patients opting for surgery [2,4,7]. A review of ABC in the jaw found that 21.8% of patients treated surgically experienced recurrence, all of whom underwent intralesional curettage [7]. The high recurrence rate of jaw ABCs may be related to the complex anatomical characteristics of the jaw and incomplete curettage. In our case, the cortical bone was disrupted due to the expansion of the lesion, making complete curettage difficult. Therefore, en bloc resection through a radical hemimandibulectomy was deemed appropriate.

However, recent studies have highlighted the efficacy of percutaneous sclerotherapy as an alternative treatment option [1-3,12]. Sclerosants cause damage to the vascular endothelium, promoting thrombus formation in the small vessels and ultimately initiating a series of processes leading to tissue repair [1]. Polidocanol is considered a safe and effective sclerosant for ABC with minimal side effects [1]. Bavan et al. reported a failure or recurrence rate of 14.7% for sclerotherapy, which is comparable to the recurrence rate for intralesional curettage [12]. In a randomized controlled trial comparing sclerotherapy with polidocanol and surgery, the treatment success rate of sclerotherapy was slightly higher (93.3%) than that of surgery (84.8%) [13]. The study also reported that patients who underwent polidocanol sclerotherapy demonstrated better post-treatment functional outcomes than those in the surgical group. Adverse events observed included local induration, skin hyperpigmentation, and dizziness, all of which were reversible and did not require medical intervention [13]. This finding suggests that sclerotherapy is a promising alternative to surgery. Although no study has documented the use of sclerotherapy using polidocanol for ABC in the jaw, we opted for this approach to avoid the functional and aesthetic impairment associated with hemimandibulectomy. After two sessions of sclerotherapy with polidocanol, the lesion showed a slight reduction, and regeneration of the surrounding cortical bone was observed. In our case, intralesional curettage within the lesion was performed after sclerotherapy according to the patient's preference. Jasper et al. reported that additional curettage was performed in 17% of 70 patients who underwent sclerotherapy using polidocanol [18]. Depending on the course of sclerotherapy, the addition of curettage to the treatment can be considered a viable option.

The application of percutaneous sclerotherapy for ABC in the jaw in our patient presented several limitations. First, the optimal number of injections and intervals for sclerotherapy in patients with ABC have not yet been established. Bavan et al. reported that the number of injections and intervals for sclerotherapy varied across studies, with an average number ranging from 1.1 to 6.4 [12]. Second, exposure of the lesion to the oral cavity led to an infection after the second injection, underscoring the importance of considering the anatomical peculiarities of the jawbone. Third, the patient’s preference for intralesional curettage after the second sclerotherapy session shortened the follow-up period. The signs of healing after sclerotherapy typically take two-three months from the initial injection and progress from several months to years after the final treatment [3]. As a result, the full therapeutic effect of sclerotherapy alone could not be adequately observed. Nevertheless, sclerotherapy promoted marginal calcification, making curettage possible. The specimen excised after sclerotherapy revealed minimal giant cell lesions, with most of the tissue replaced by fibrous connective tissue and bone tissue. These findings may suggest changes after sclerotherapy.

## Conclusions

In conclusion, we successfully treated a rapidly growing ABC in the mandible of a teenage girl using a combination of polidocanol sclerotherapy and intralesional curettage. Sclerotherapy using polidocanol-induced enhanced calcification around the lesion, enabling intralesional curettage. This approach preserved the jawbone and avoided functional and aesthetic impairments associated with en bloc resection. Given the high recurrence rate associated with intralesional curettage and the functional and aesthetic impacts of en bloc resection in jaw ABC, percutaneous sclerotherapy with polidocanol emerges as a promising option. Further investigation is needed to determine the optimal application, number of injections, intervals, and follow-up periods for percutaneous sclerotherapy with polidocanol in the jawbone.
